# ﻿An enigmatic new octocoral species (Anthozoa, Octocorallia, Malacalcyonacea) from Isla del Coco National Park

**DOI:** 10.3897/zookeys.1169.100576

**Published:** 2023-07-18

**Authors:** Odalisca Breedy, Catherine S. McFadden, Jorge Cortés

**Affiliations:** 1 Centro de Investigación en Ciencias del Mar y Limnología; Museo de Zoología, Centro de Investigación en Biodiversidad y Ecología Tropical, Universidad de Costa Rica, P.O. Box 11501-2060, San José, Costa Rica; 2 Centro de Investigación en Estructuras Microscópicas, Universidad de Costa Rica, San José, Costa Rica; 3 Smithsonian Tropical Research Institute, Panama, Panama; 4 Department of Biology, Harvey Mudd College, Claremont, CA 91711-5990, USA

**Keywords:** Biodiversity, new genus, new species, oceanic island, soft corals, taxonomy

## Abstract

*Alienaparva***gen. et sp. nov.** is described from Cocos Island, Costa Rica. The species was found at various islets and rocky outcrops north and northwest of the island, 20–30 m in depth. The genus is characterised by polyps, retracting into calyces, that form thin encrusting mats extending on dead or live substrates. Sclerites are mostly asymmetrical spindles. Anthocodial rods are arranged in points, not forming a collaret. Colonies and coenenchymal sclerites are red, and polyps are transparent. Using an integrative taxonomic approach, we found the new genus to morphologically and genetically differ from all other described taxa. The molecular phylogenetic analyses provide strong support for the placement of this new genus in the family Pterogorgiidae. Morphologically it is unlike any of the other members of this family, necessitating an amendment to the diagnosis of Pterogorgiidae. Like several other known taxa of octocorals with encrusting growth forms, *Aliena***gen. nov.** appears to have evolved from a gorgonian ancestor by loss of an internal skeletal axis. It is the first member of Pterogorgiidae to be reported from the eastern Pacific, contributing further to the knowledge of marine biodiversity in the eastern tropical Pacific and to the octocoral biodiversity of Cocos Island in particular.

## ﻿Introduction

The occurrence of shallow-water octocorals in Isla del Coco National Park is rare in comparison to other oceanic islands, like the Galápagos Islands (Ecuador) or the Revillagigedo Archipelago (México), where the number of reported species is higher (Williams and Breedy 2004; Bedolla 2007; Breedy and Cortés 2008, 2011; Hickman 2008; Breedy et al. 2009; Olvera et al. 2018). The reason for this difference is still unknown, but it could simply be that more exploration is needed. The octocoral diversity in Isla del Coco increases towards mesophotic depths (Breedy et al. 2012, 2021; Cortés 2019), and the fauna is different from that of the shallow communities. Presently, five species have been reported from 10 to 35 m in depth: the gorgoniids *Leptogorgiaalba* Verrill, 1868, *Leptogorgiatricorata* Breedy & Cortés, 2008, and *Pacifigorgiacurta* Breedy & Guzman, 2003, and two stoloniferans, *Carijoariisei* (Duchassaing & Michelotti, 1860) and *Rhodoliticaocculta* Breedy, McFadden, Murillo & Vargas, 2021. For the time being, three of them are considered endemic to Isla del Coco.

Recently, a new octocoral was collected and photographed at several sites at the northern part of the Island. Herein, we describe a new genus and species using an integrative taxonomic approach, combining morphological and molecular analyses to phylogenetically position this monospecific genus within Octocorallia. This study is a contribution to the knowledge of the octocoral biodiversity of Isla del Coco and marine biodiversity of the eastern tropical Pacific oceanic islands.

## ﻿Materials and methods

### ﻿Study site and collection methods

**Figure 1.**
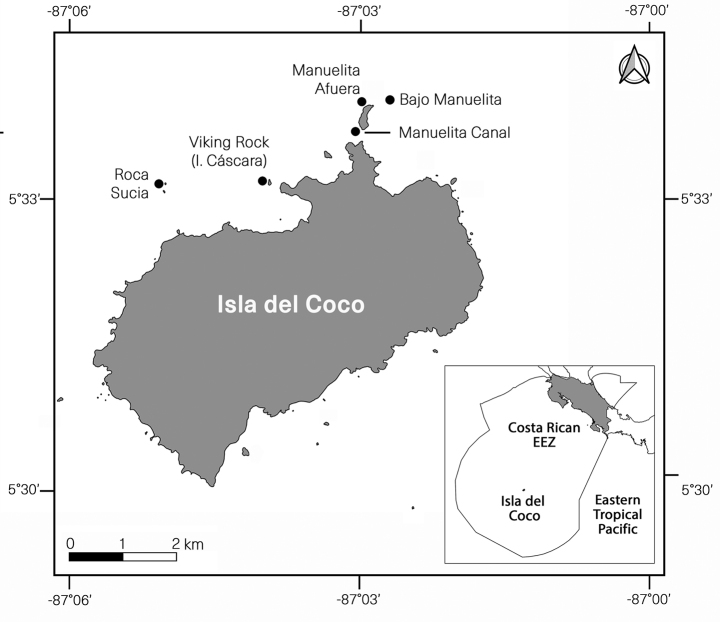
Map showing the locations where *Alienaparva* gen. nov. et sp. nov. was found at Isla del Coco, Costa Rica. (Map by Beatriz Naranjo, University of Costa Rica.)

Isla del Coco National Park is an oceanic island located between 5°30'–5°34'N and 87°01'–87°06'W in the eastern Tropical Pacific approximately 500 km southwest of Costa Rica and more than 600 km northeast of the Galápagos Islands, Ecuador (Cortés 2016; Breedy et al. 2021) (Fig. 1). The specimens were collected by scuba diving at depths down to 30 m from various points north and northwest of the Island. The colonies were observed and photographed *in situ* during four different trips: August 2021, October 2021, December 2021, and January 2022. Samples were collected and preserved in 95% ethanol for further analyses. The holotype and paratypes are deposited at the Zoology Museum, University of Costa Rica, Costa Rica (**MZUCR**).

### ﻿Morphological analysis

For taxonomic identification, external characters of the colony were analysed from the *in situ* photographs and dissection of collected samples under a stereoscope. For internal characters, sclerites from polyps and coenenchyme were obtained by dissolving the tissue in 5% sodium hypochlorite; dissociated sclerites were washed several times in distilled water until organic matter was completely removed, dehydrated with 100% ethanol, and subsequently dried in an oven. Sclerites were prepared for light microscopy, mounted in glycerine, and photographed with an Olympus LX 51 inverted microscope. For scanning electron microscopy (SEM), sclerites were mounted on SEM stubs by double stick carbon tape and silver paint, then sputter-coated with gold, 30–60 nm layer, in EMS 550X Ion Coater; the images were obtained using a FESEM Zeiss Sigma 300 and a Hitachi NSEM 3700 (at 15kV). Measurements of the sclerites were obtained from optical and SEM images. Taxonomic terminology follows Bayer et al. (1983) for sclerites and McFadden et al. (2022).

### ﻿Molecular phylogenetic analysis

DNA was isolated from ethanol-preserved tissue of two specimens using the modified salting-out protocol of Herrera (2022). The mitochondrial *mtMutS* and nuclear *28S rDNA* markers commonly used for DNA barcoding of octocorals were amplified using PCR with previously published primers (ND4-2599F and mut3458R for *mtMutS*; 28S-Far and 28S-Rar for *28S rDNA*) and protocols (McFadden et al. 2011; McFadden and van Ofwegen 2013). Amplicons were purified by PEG-precipitation (Sánchez et al. 2003) and Sanger-sequenced. Sequences for *mtMutS* were aligned by eye to the reference alignment of McFadden et al. (2022). This alignment includes representatives of all genera of octocorals for which *mtMutS* sequences are available. Preliminary maximum-likelihood (ML) phylogenetic analyses using PhyML (Guindon and Gascuel 2003) supported the placement of the new species in Order Malacalcyonacea. Further phylogenetic analyses of *mtMutS* were conducted using just the subset of the alignment that included taxa from that order. Sequences for *28S rDNA* were aligned to a reference dataset that included as many as possible of the same individuals that were included in the *mtMutS* alignment. *28S rDNA* sequences were not available for 23% of the taxa in the *mtMutS* dataset, and for 9% of the taxa we included a *28S rDNA* sequence that was from a different specimen of the same species (Suppl. material 1). *28S rDNA* sequences were aligned using MAFFT (Katoh et al. 2005) and subsequently trimmed internally to remove regions of poor alignment using gblocks (Castresana 2000) as implemented at Phylogeny.fr (Dereeper et al. 2008).

Phylogenetic analyses were run separately for each alignment. ML analyses were run using IQTree v. 2.1.2 (Nguyen et al. 2015) with the model of evolution (TVM+F+R5 for *mtMutS*; GTR+F+R4 for *28S rDNA*) selected by ModelFinder (Kalyaanamoorthy et al. 2017) and support from 1000 ultrafast bootstrap replicates (Hoang et al. 2018). MrBayes v. 3.2 (Ronquist et al. 2012) was used for Bayesian phylogenetic reconstruction using a GTR+I+G model of evolution. MrBayes was run for 8 × 10^6^ (*mtMutS*) or 5 × 10^6^ (*28S rDNA*) generations (until standard deviation of split partitions <0.01) with a 25% burnin and default Metropolis coupling parameters.

## ﻿Results

### ﻿Systematics

**SubPhylum Anthozoa Ehrenberg, 1831**

**Class Octocorallia Haeckel, 1866**

**Order Malacalcyonacea McFadden, van Ofwegen & Quattrini, 2022**

**Family Pterogorgiidae McFadden, van Ofwegen & Quattrini, 2022**

#### ﻿Genus Aliena gen. nov.

Taxon classificationAnimaliaMalacalcyonaceaPterogorgiidae

100CA82B-4A83-59D7-9942-FC1CFE5B389F

https://zoobank.org/4A8773BF-3BC3-479E-9C13-1547DF74D2C7

##### Diagnosis.

Colonies are encrusting mats of irregular shape, consisting of clusters of 3–25 polyps that may be interconnected by thin coenenchymal extensions forming bridge-like bands between them. Colonies lack an axis. Polyps are retractile into calyces that are covered by a dense layer of small sclerites producing a granular appearance. Coenenchyme is thin without differential sclerite layers. Coenenchymal sclerites are mostly red asymmetrical spindles with simple tubercles and irregular ends. Anthocodial sclerites are mostly red flat rods, with serrated or prickly borders and sparse thorns on the surface, and spine-like rods. They are arranged “*en chevron*”, forming points but not a collaret. Flat rods form longitudinal rows along the polyp body. Tentacular sclerites are mostly yellow, biscuit-like rods. Colonies are dark red in life and maintain that colour in ethanol.

##### Type species.

*Alienaparva* sp. nov. by original designation.

##### Etymology.

*Alienus* (L) foreign, strange, not related. The generic name refers to the unexpected appearance or unnoticed presence of a new shallow-water taxon. It also alludes to its surprising phylogenetic relationship to a group of gorgonian octocorals, a relationship not predicted by its morphology. Gender feminine.

#### ﻿ Aliena parva sp. nov.

Taxon classificationAnimaliaMalacalcyonaceaPterogorgiidae

59BD0382-446A-56DE-8A88-09DCCAEE1184

https://zoobank.org/11BAFF5A-A43A-46E3-AB48-95FB6986EE27

**Figure 2.**
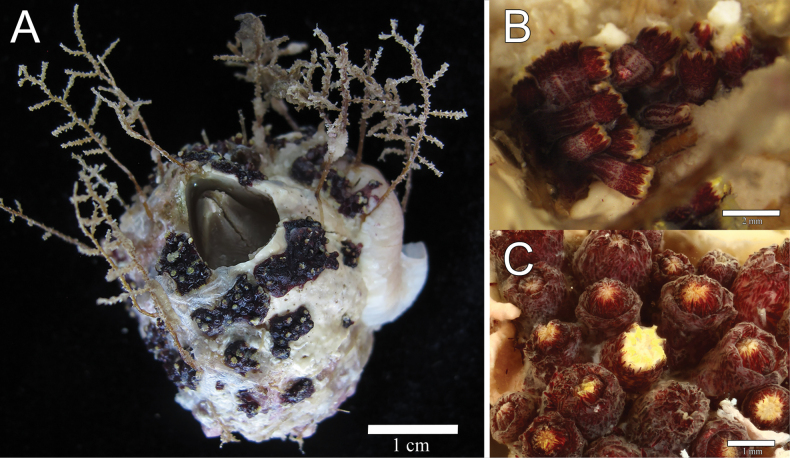
*Alienaparva* gen. nov. et sp. nov. **A** holotype, MZUCR 3679 **B** polyps, partially retracted showing anthocodial sclerites **C** polyp mounds. (Photographs by Fiorella Vásquez, University of Costa Rica.)

**Figure 3.**
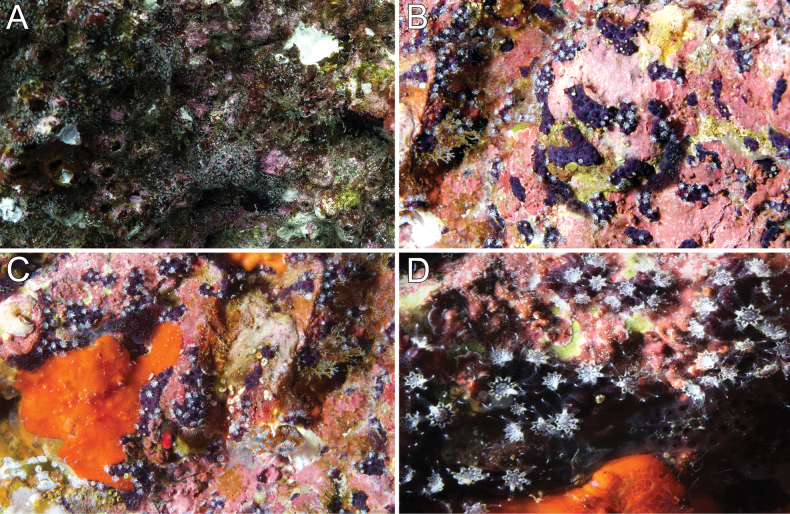
Colonies *in situ*. **A** Manuelita Afuera, panoramic view of the wall, 25 m deep. (photograph by Anuar Patjane) **B–D** Manuelita Canal, 25 m deep (photographs by Avi Klapfer).

**Figure 4.**
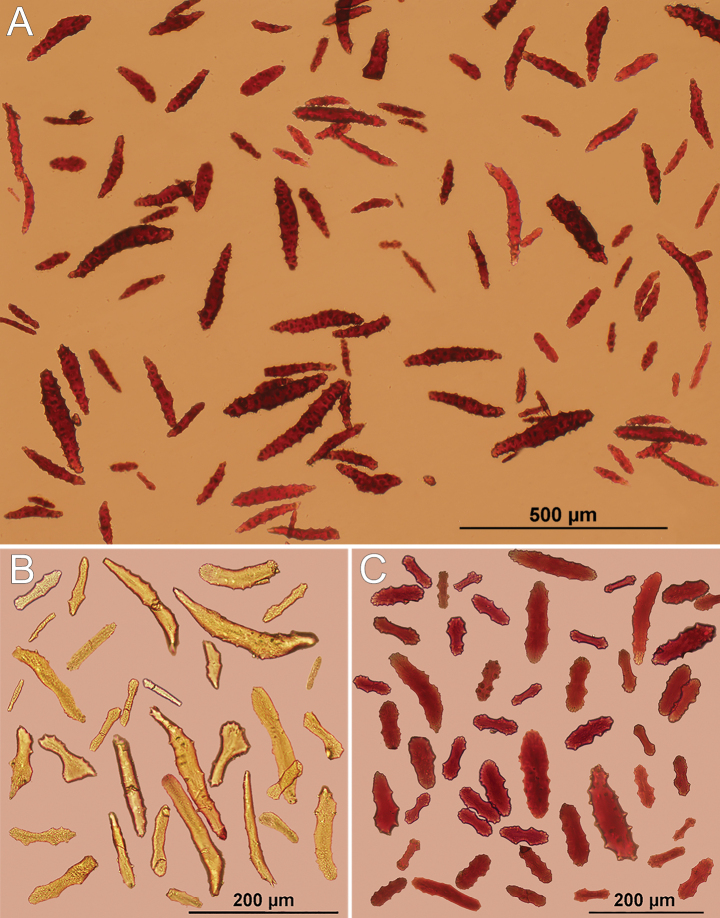
*Alienaparva* gen. nov. et sp. nov., holotype MZUCR 3679 sclerites. **A** unsorted coenenchymal sclerites **B** tentacular sclerites **C** anthocodial sclerites.

**Figure 5.**
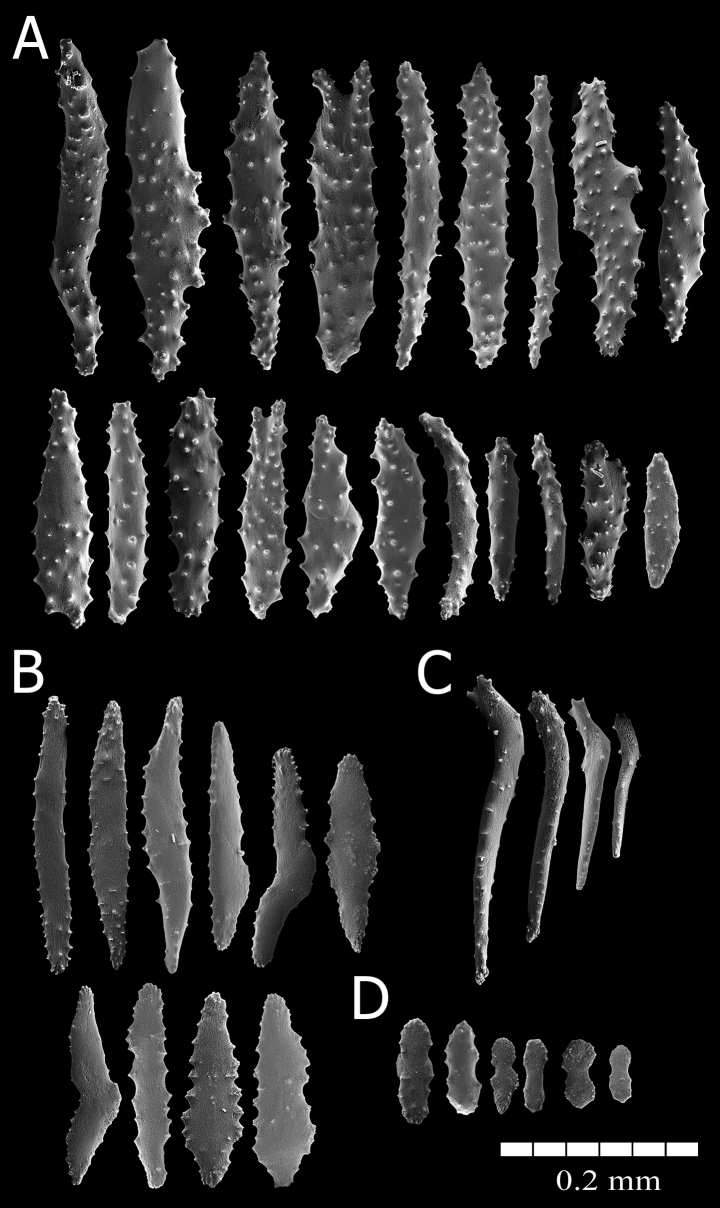
*Alienaparva* gen. nov. et sp. nov., holotype MZUCR 3679 SEM sclerites. **A** coenenchymal sclerites **B, D** anthocodial sclerites **C** tentacular sclerites.

**Figure 6.**
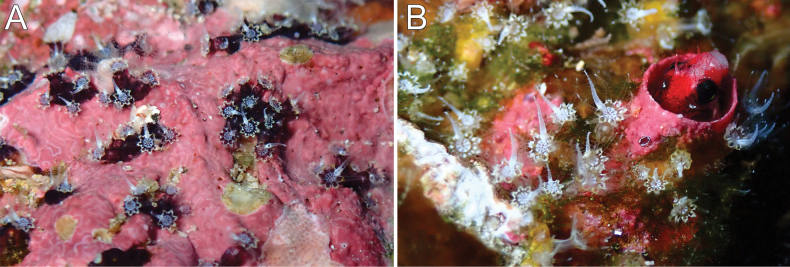
Colonies *in situ* with modified tentacle, January 2022. **A** Manuelita Canal, 25–30 m deep **B** Roca Sucia, 25 m deep.

Figs 2 , 3 , 4 , 5 , 6

##### Materials examined.

***Holotype***. MZUCR 3679, lot 1, ethanol-preserved, Manuelita Afuera, Isla del Coco, 05°33.791'N, 087°02.934'W, 22 m depth, J. Cortés and M. Cruz, 4 December 2021. ***Paratypes*.**MZUCR 3680, lot 2, same data as holotype. MZUCR 3681, lot 1, lot 2, ethanol-preserved, Manuelita Canal, Isla del Coco, 05°33.524'N, 087°02.940'W, 20–30 m depth, B. Naranjo, 12 October 2021. MZUCR 3682, ethanol-preserved, Bajo Manuelita, Isla del Coco, 05°33.849'N, 087°02.676'W, 23 m, J. Cortés and A. Klapfer, 6 December 2021. MZUCR 3683, Manuelita Afuera, Isla del Coco, 05°33.791'N, 087°02.934'W, 29 m depth, O. Breedy, 28 September 2022. MZUCR 3684, Manuelita Afuera, Isla del Coco, 05°33.791'N, 087°02.934'W, 25 m depth, O. Breedy, 4 October 2022.

##### Type locality.

Isla del Coco, Pacific Costa Rica, at depths of 20–30 m.

##### Description.

The holotype is formed of 15 scattered clusters of polyps encrusting the surface of a barnacle about 4 cm in diameter; the barnacle plates are covered by many epibionts and several small, unbranched hydroids (Fig. 2A). The polyps are in clusters, 0.15–1.10 cm in longest dimension and composed of 3–20 polyps (Fig. 2A). The polyps are closely spaced; those that are preserved partially expanded are up to 2.0 mm tall (from the base to the proximal border of tentacles) (Fig. 2B). Polyps are retractile into calyces that are up to 0.75 mm in diameter, and up to 1.0 mm tall when the anthocodia is retracted. The surface of the calyx is covered by a dense layer of sclerites giving it a slightly granular appearance. The tentacles are transparent with yellow sclerites. When retracted, the yellow sclerites can be observed at the polyp-mound summit (Fig. 2C). The coenenchyme is thin and without differential sclerite layers; it extends over the substrate. Coenenchymal sclerites are asymmetrical spindles with variable ends: pointed, blunt, bifurcated, or a combination (Figs 4, 5). They are straight or slightly curved, 0.16–0.35 mm long, and 0.02–0.08 mm wide (Figs 4A, 5A), with simple tubercles, not very crowded on the surface; they do not have complex tubercles or waists. Anthocodial sclerites are mostly red flat rods, 0.20–0.30 mm long and 0.02–0.06 mm wide, with serrated or prickly borders and sparse thorns on the surface, and smaller biscuit-like rods, 0.06–0.10 mm long and 0.02–0.03 mm wide (Fig. 4C, 5B). Anthocodial rods are arranged ‘*en chevron*’, forming points but not a collaret (Fig. 2B). The flat rods are arranged in longitudinal rows along the polyp body (Fig. 2B, 5D). Tentacular sclerites are spine-like rods with a bent end 0.14–0.29 mm long and 0.02–0.03 mm wide (Figs 4B, 5C).

Colonies are dark red in life and when preserved (Fig. 2A–C). Coenenchymal sclerites are mostly dark red, but sometimes with lighter hues (Fig. 4A). The characteristics of the paratypes are very consistent with those of the holotype.

##### Remarks.

The colonies are overgrowing dead or live substrates, encrusting small rocks, barnacle plates, shells, or among turf. They were frequently found among the worm tubes occupied by the endemic fish *Acanthemblemariaatrata* Hastings & Robertson, 1999 from Isla del Coco (Figs 3C, 6B). When polyps are fully expanded, the gastric cavities of the polyps extend high over the polyp mounds, and the oral disk prolongs into eight rays marked by small, red rods along the intertentacular margins (Figs 3B–D, 6A). We noticed during the January 2022 and September-October 2022 trips that the polyps have one tentacle that is opaque and appears to be somewhat swollen at its base (Fig. 6A, B). This differentiated tentacle was not present in the colonies observed during the other trips in 2021. Reasons for this difference and possible functions of this differentiated tentacle remain unknown.

##### Distribution.

Colonies were found at various localities north of Isla del Coco around Manuelita Afuera, Manuelita Canal and Bajo Manuelita; and northwest at Roca Sucia, 05°32.875'N, 087°04.956'W and Viking Rock (Isla Cáscara), 05°33.006'N, 087°03.865'W, NW of the island (Fig. 1). Only known from the type locality. The bathymetric range was 20–30 m.

##### Etymology.

*Parvus* (L), in allusion to the small size of the polyps. Gender feminine: *parva*.

### ﻿Phylogenetic analysis

**Figure 7.**
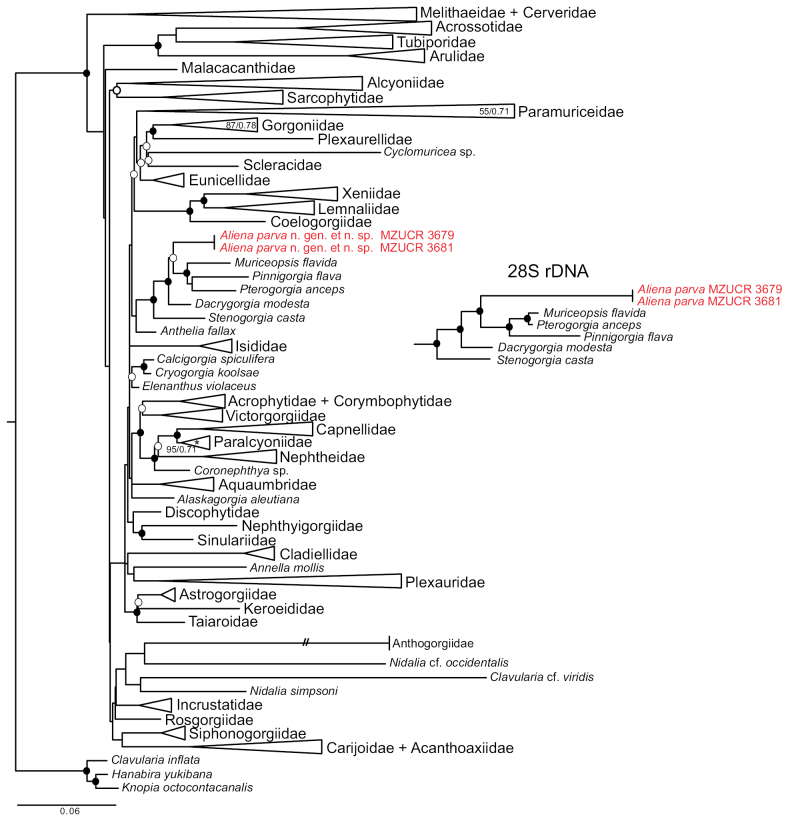
Maximum-likelihood tree of Malacalcyonacea based on *mtMutS*. Support values indicated by symbols at nodes. Black circles: maximum-likelihood bootstrap value (bs) > 70% and Bayesian posterior probability (pp) > 0.9); open circles: bs > 70, no support (pp <0.9) from Bayesian analysis. Families have been collapsed to facilitate readability. All collapsed clades have bs > 70% and pp > 0.9 unless otherwise noted. The branch leading to Anthogorgiidae has been shortened to fit the page. Inset shows Pterogorgiidae clade from analysis of *28S rDNA*.

Phylogenetic analyses of both the mitochondrial *mtMutS* and nuclear *28S rDNA* genes strongly supported the placement of *Alienaparva* gen. nov. et sp. nov. in the octocoral family Pterogorgiidae (Fig. 7). All analyses supported *A.parva* as the sister to a clade of Pterogorgiidae that includes the gorgonian genera *Pterogorgia* Ehrenberg, 1834, *Pinnigorgia* Grasshoff & Alderslade, 1997, and *Muriceopsis* Aurivillius, 1931. Many of the relationships among and within other families of Malacalcyonacea were only very poorly supported by the *28S rDNA*ML tree (Suppl. material 2) and by both Bayesian trees (Suppl. materials 3, 4), and differed from the *mtMutS*ML tree. Nonetheless, all phylogenetic analyses of both genes independently offered strong support (bootstrap values >99%; posterior probabilities >0.9) for the monophyly of Pterogorgiidae and the position of *A.parva* within that clade (Fig. 7). The moderately long branch length separating *A.parva* from other genera in Pterogorgiidae in both *mtMutS* and *28S rDNA* trees further supports its status as a new genus.

## ﻿Discussion

The external morphology of *Aliena* gen. nov. suggests it could belong to one of the families of stoloniferous octocorals, several of which (e.g., Carijoidae, Incrustatidae, Sarcodictyonidae, Tubiporidae) include genera of encrusting colonies that produce mats extending on hard substrata (McFadden et al. 2022). It bears a particular superficial resemblance to some of the genera in family Tubiporidae such as *Stragulum* van Ofwegen & Haddad, 2011, but all members of that family have sclerites in either the coenenchyme or polyp body wall that are fused. The molecular evidence from both a mitochondrial (*mtMutS*) and nuclear (*28S rDNA*) gene, however, suggest that *Aliena* gen. nov. does not belong to any of the families that comprise mostly stoloniferous taxa, but instead strongly support its placement in the gorgonian family Pterogorgiidae.

The gross colony morphology of *Aliena* gen. nov. is unlike any of the other genera in Pterogorgiidae, all of which have internal skeletal axes of gorgonin that support an erect growth form. Its sclerome is, however, reasonably consistent with that of the other Pterogorgiidae, all of which have asymmetrically spiny or curved spindles in the coenenchyme and flattened rods in the polyps (McFadden et al. 2022). The polyp sclerites of *Aliena* gen. nov. are similar in particular to those of *Pterogorgia* and *Stenogorgia* Verrill, 1883, which are usually small spindles or flattened rods that are occasionally arranged as collaret and points or just points as in *Aliena* gen. nov.

*Aliena* gen. nov. lacks the additional sclerite forms that are typical of some of the other genera of Pterogorgiidae, such as the capstans of *Pterogorgia* and *Stenogorgia* or the balloon clubs of *Dacrygorgia* McFadden, van Ofwegen & Quattrini, 2022. The simple thorn-like ornaments on the coenenchymal spindles of *Aliena* gen.nov. also differ markedly from the asymmetrical spines and high, complex tubercles that characterize the spindles of *Muriceopsis* and *Tobagogorgia* Sánchez, 2007.

*Aliena* gen. nov. is one of several genera of octocorals with a simple, encrusting growth form that belong to clades whose other members are all gorgonians, i.e., species with an internal skeletal axis of proteinaceous or calcareous material. Other examples include the encrusting genera *Thrombophyton* McFadden & Hochberg, 2003, which falls within the gorgonian family Paramuriceidae, and *Discophyton* McFadden & Hochberg, 2003 (recently assigned to the monotypic family Discophytidae), which belongs to a larger clade that consists almost exclusively of gorgonians (McFadden et al. 2022). The most parsimonious interpretation of these surprising phylogenetic relationships is that these three genera have evolved independently from gorgonian ancestors by the secondary loss of an axis, a scenario that has been supported by ancestral state reconstruction of skeletal evolution in Octocorallia (Quattrini et al. 2020).

Placement of *Aliena* gen. nov. in Pterogorgiidae requires amending the diagnosis of that family to accommodate a species that lacks an axis (changes in bold):

### ﻿Diagnosis (modified from McFadden et al. 2022)

Octocorals with (**or rarely without**) a proteinaceous skeletal axis. Axis hollow with wide, cross-chambered central core. Colonies **encrusting or** erect, sparsely to profusely branched (dichotomous, pinnate), planar or bushy; branches may be flattened, oval or triangular in cross-section. Polyps monomorphic, retractile into coenenchyme or into low calyces, distributed evenly over branch surface or arranged biserially in recessed grooves along branch margins. Polyp sclerites small, flattened rods or slender spindles only rarely arranged as collaret and points. Sclerites of coenenchyme **typically** include asymmetrically spiny or curved spindles with or without complex tubercular ornamentation; capstans, asymmetrical clubs or balloon-clubs, double-heads or plates may also be present. Zooxanthellate or azooxanthellate.

*Aliena* gen. nov. is the second genus of Pterogorgiidae known to have a distribution in the Pacific, and the only one recorded so far from the eastern Pacific. The majority of the taxa in this family are distributed in the tropical Atlantic.

## Supplementary Material

XML Treatment for Aliena

XML Treatment for Aliena parva

## ﻿Additional information

### Author ORCIDs

Odalisca Breedy https://orcid.org/0000-0001-5686-4164

Catherine S. McFadden https://orcid.org/0000-0002-8519-9762

Jorge Cortés https://orcid.org/0000-0001-7004-8649

### Data availability

All of the data that support the findings of this study are available in the main text or Supplementary Information.

## Supplementary materials

Supplementary material 1 Sequences included in molecular phylogenetic analyses of Order Malacalcyonacea Data type Phylogenetic https://binary.pensoft.net/file/880670This dataset is made available under the Open Database License (http://opendatacommons.org/licenses/odbl/1.0/). The Open Database License (ODbL) is a license agreement intended to allow users to freely share, modify, and use this Dataset while maintaining this same freedom for others, provided that the original source and author(s) are credited.Odalisca Breedy, Catherine S. McFadden, Jorge Cortés

Supplementary material 2 Phylogenetic relationships of Order Malacalcyonacea based on maximum likelihood analysis of 28S rDNA Data type Phylogenetic Explanation note Numbers on branches are bootstrap percentages from 1000 ultrafast bootstrap replicates. https://binary.pensoft.net/file/880671This dataset is made available under the Open Database License (http://opendatacommons.org/licenses/odbl/1.0/). The Open Database License (ODbL) is a license agreement intended to allow users to freely share, modify, and use this Dataset while maintaining this same freedom for others, provided that the original source and author(s) are credited.Odalisca Breedy, Catherine S. McFadden, Jorge Cortés

Supplementary material 3 Phylogenetic relationships of Order Malacalcyonacea based on Bayesian analysis of mtMutS Data type Phylogenetic Explanation note Numbers on branches are posteriori probabilities. https://binary.pensoft.net/file/880672This dataset is made available under the Open Database License (http://opendatacommons.org/licenses/odbl/1.0/). The Open Database License (ODbL) is a license agreement intended to allow users to freely share, modify, and use this Dataset while maintaining this same freedom for others, provided that the original source and author(s) are credited.Odalisca Breedy, Catherine S. McFadden, Jorge Cortés

Supplementary material 4 Phylogenetic relationships of Order Malacalcyonacea based on Bayesian analysis of 28S rDNA Data type Phylogenetic Explanation note Numbers on branches are posteriori probabilities. https://binary.pensoft.net/file/880673This dataset is made available under the Open Database License (http://opendatacommons.org/licenses/odbl/1.0/). The Open Database License (ODbL) is a license agreement intended to allow users to freely share, modify, and use this Dataset while maintaining this same freedom for others, provided that the original source and author(s) are credited.Odalisca Breedy, Catherine S. McFadden, Jorge Cortés

## References

[B1] Aurivillius M (1931) The gorgonians from Dr. Sixten Bock’s expedition to Japan and the Bonin Islands, 1914. Kungliga Svenska Vetenskapsakademiens Handlingar (Serie 3) 9(4): 1–337.

[B2] Bayer FM Grasshoff M Verseveldt J (1983) Illustrated Trilingual Glossary of Morphological and Anatomical Terms Applied to Octocorallia. E.J. Brill/Dr. W. Backhuys, Leiden, 75 pp.

[B3] Bedolla Y (2007) Caracterización Ecológica de la Comunidad de Macroinvertebrados Marinos Submareales Rocosos del Archipiélago de Revillagigedo, México. Bachaelor’s thesis, Universidad Autónoma de Baja California Sur, La Paz, 121 pp.

[B4] Breedy O Cortés J (2008) Octocorals (Coelenterata: Anthozoa: Octocorallia) of Isla del Coco, Costa Rica. Revista de Biología Tropical 56(Supplement 2): 71–77.

[B5] Breedy O Cortés J (2011) Morphology and taxonomy of a new species of *Leptogorgia* (Cnidaria: Octocorallia: Gorgoniidae) in Cocos Island National Park, Pacific Costa Rica. Proceedings of the Biological Society of Washington 124(2): 62–69. 10.2988/10-18.1

[B6] Breedy O Guzman HM (2003) Octocorals from Costa Rica: the genus *Pacifigorgia* (Coelenterata: Octocorallia: Gorgoniidae). Zootaxa 281(1): 1–60. 10.11646/zootaxa.281.1.1

[B7] Breedy O Hickman Jr CP Williams GC (2009) Octocorals in the Galapagos Islands. Galapagos Research 66: 27–31.

[B8] Breedy O van Ofwegen LP Vargas S (2012) A new family of soft corals (Anthozoa, Octocorallia, Alcyonacea) from the aphotic tropical eastern Pacific waters revealed by integrative taxonomy. Systematics and Biodiversity 10(3): 351–359. 10.1080/14772000.2012.707694

[B9] Breedy O van Ofwegen LP McFadden CS Murillo-Cruz C (2021) *Rhodolitica* on rhodoliths: A new stoloniferan genus (Anthozoa, Octocorallia, Alcyonacea). ZooKeys 1032: 63–77. 10.3897/zookeys.1032.63431 33958916 PMC8065023

[B10] Castresana J (2000) Selection of conserved blocks from multiple alignments for their use in phylogenetic analysis. Molecular Biology and Evolution 17(4): 540–552. 10.1093/oxfordjournals.molbev.a026334 10742046

[B11] Cortés J (2016) Isla del Coco: coastal and marine ecosystems. In: Kappelle M (Ed.) Costa Rican Ecosystems.University of Chicago Press, Chicago/London, 162–191. 10.7208/chicago/9780226121642.003.0007

[B12] Cortés J (2019) Isla del Coco, Costa Rica, Eastern Tropical Pacific. In: Loya Y Puglise KA Bridge TCL (Eds) Mesophotic Coral Ecosystems.Coral Reefs of the World 12. Springer Nature, Cham, 465–474. 10.1007/978-3-319-92735-0_26

[B13] Dereeper A Guignon V Blanc G Audic S Buffet S Chevenet F Dufayard JF Guindon S Lefort V Lescot M Claverie JM Gascuel O (2008) Phylogeny.fr: Robust phylogenetic analysis for the non-specialist. Nucleic Acids Research 36(Web Server): W465–W469. 10.1093/nar/gkn180 PMC2447785 18424797

[B14] Duchassaing P Michelotti G (1860) Mémoire sur les coralliaires des Antilles. Memorie della Reale Accademia delle Scienze di Torino (Serie 2) 19: 279–365. 10.5962/bhl.title.11388

[B15] Ehrenberg CG (1831) Symbolae physicae, seu icones et descriptiones corporum naturalium novorum. Pars Zoologica, 4, Berlin.

[B16] Ehrenberg CG (1834) Beiträge zur physiologischen Kenntniss der Corallenthiere im allgemeinen, und besonders des rothen Meeres, nebst einem Versuche zur physiologischen Systematik derselben. Abhandlungen der Königlichen Akademie der Wissenschaften, Berlin 1: 225–380. https://www.biodiversitylibrary.org/page/2972586

[B17] Grasshoff M Alderslade P (1997) Gorgoniidae of Indo-Pacific reefs with descriptions of two new genera (Coelenterata: Octocorallia). Senckenbergiana Biologica 77: 23–25.

[B18] Guindon S Gascuel O (2003) A simple, fast, and accurate algorithm to estimate large phylogenies by maximum likelihood. Systematic Biology 52(5): 696–704. 10.1080/10635150390235520 14530136

[B19] Haeckel E (1866) Generelle Morphologie der Organismen. G. Reimer, Berlin, 1036 pp. 10.1515/9783110848281

[B20] Herrera S (2022) Salting-out protocol for extracting HMW genomic DNA from frozen octocorals. 10.17504/protocols.io.bypypvpw

[B21] Hickman Jr CP (2008) A Field Guide to Corals and Other Radiates of Galapagos. Sugar Spring Press, Lexington, 162 pp.

[B22] Hoang DT Chernomor O von Haeseler A Minh BQ Vinh LS (2018) UFBoot2: Improving the ultrafast bootstrap approximation. Molecular Biology and Evolution 35(2): 518–522. 10.1093/molbev/msx281 29077904 PMC5850222

[B23] Kalyaanamoorthy S Minh BQ Wong TKF Haeseler A Jermiin LS (2017) ModelFinder: Fast model selection for accurate phylogenetic estimates. Nature Methods 14(6): 587–589. 10.1038/nmeth.4285 28481363 PMC5453245

[B24] Katoh K Kumar KI Toh H Miyata T (2005) MAFFT version 5: Improvement in accuracy of multiple sequence alignment. Nucleic Acids Research 33(2): 511–518. 10.1093/nar/gki198 15661851 PMC548345

[B25] McFadden CS Hochberg FG (2003) Biology and taxonomy of encrusting alcyoniid soft corals in the northeastern Pacific Ocean with descriptions of two new genera (Cnidaria, Anthozoa, Octocorallia). Invertebrate Biology 122(2): 93–113. 10.1111/j.1744-7410.2003.tb00076.x

[B26] McFadden CS van Ofwegen LP (2013) A second, cryptic species of the soft coral genus *Incrustatus* (Anthozoa: Octocorallia: Clavulariidae) from Tierra del Fuego, Argentina revealed by DNA barcoding. Helgoland Marine Research 67(1): 137–147. 10.1007/s10152-012-0310-7

[B27] McFadden CS Benayahu Y Pante E Thoma JN Nevarez PA France SC (2011) Limitations of mitochondrial gene barcoding in Octocorallia. Molecular Ecology Resources 11(1): 19–31. 10.1111/j.1755-0998.2010.02875.x 21429097

[B28] McFadden CS van Ofwegen LP Quattrini AM (2022) Revisionary systematics of Octocorallia (Cnidaria: Anthozoa) guided by phylogenomics. Bulletin of the Society of Systematic Biologists 1(3): е8735. 10.18061/bssb.v1i3.8735

[B29] Nguyen L-T Schmidt HA von Haeseler A Minh BQ (2015) IQ-TREE: A fast and effective stochastic algorithm for estimating maximum likelihood phylogenies. Molecular Biology and Evolution 32(1): 268–274. 10.1093/molbev/msu300 25371430 PMC4271533

[B30] Olvera U Hernández O Sánchez C Gómez-Gutiérrez J (2018) Two new endemic species of Gorgoniidae (Cnidaria, Anthozoa, Octocorallia) from Revillagigedo Archipelago, Mexico. Zootaxa 4442(4): 523–538. 10.11646/zootaxa.4442.4.2 30313949

[B31] Quattrini AM Rodríguez E Faircloth BC Cowman P Brugler MR Farfan G Hellberg ME Kitahara MV Morrison CL Paz-García DA Reimer JD McFadden CS (2020) Paleoclimate ocean conditions shaped diversification of coral skeletal composition through deep time. Nature Ecology & Evolution 4: 1531–1538. 10.1038/s41559-020-01291-1 32868916

[B32] Ronquist F Teslenko M van der Mark P Ayres D Darling A Höhna S Larget B Liu L Suchard MA Huelsenbeck JP (2012) MrBayes 3.2: Efficient Bayesian phylogenetic inference and model choice across a large model space. Systematic Biology 61(3): 539–542. 10.1093/sysbio/sys029 22357727 PMC3329765

[B33] Sánchez JA (2007) A new genus of Atlantic octocorals (Octocorallia: Gorgoniidae): systematics of gorgoniids with asymmetric sclerites. Journal of Natural History 41(9–12): 493–509. 10.1080/00222930701237315

[B34] Sánchez JA McFadden CS France SC Lasker HR (2003) Phylogenetic analyses of shallow-water Caribbean octocorals. Marine Biology 142(5): 975–987. 10.1007/s00227-003-1018-7

[B35] van Ofwegen LP Haddad MA (2011) A probably invasive new genus and new species of soft coral (Octocorallia: Alcyonacea: Clavulariidae) from Brazil. Zootaxa 3107(1): 38–46. 10.11646/zootaxa.3107.1.2

[B36] Verrill AE (1868) Notes on Radiata in the Museum of Yale College, number 6: review of the corals and polyps of the west coast of America. Transactions of the Connecticut Academy of Arts and Sciences 1 (2^nd^ edn): 377–422. [pls 4–10]

[B37] Verrill AE (1883) Report on the Anthozoa, and on some additional species dredged by the “Blake” in 1877–1879, and by the U.S. Fish Commission steamer “Fish Hawk” in 1880–82. Bulletin of the Museum of Comparative Zoology at Harvard College 11: 1–72. [pls 1–8]

[B38] Williams GC Breedy O (2004) The Panamic gorgonian genus *Pacifigorgia* (Octocorallia: Gorgoniidae) in the Galápagos Archipelago, with descriptions of three new species. Proceedings of the California Academy of Sciences 55: 55–88. https://biostor.org/reference/110172

