# Supplementary figures and images for: ﻿An enigmatic new octocoral species (Anthozoa, Octocorallia, Malacalcyonacea) from Isla del Coco National Park
Source: Zookeys. 2023 Jul 18;1169:317–31. doi: 10.3897/zookeys.1169.100576 (PMC10369172; doi:10.3897/zookeys.1169.100576)

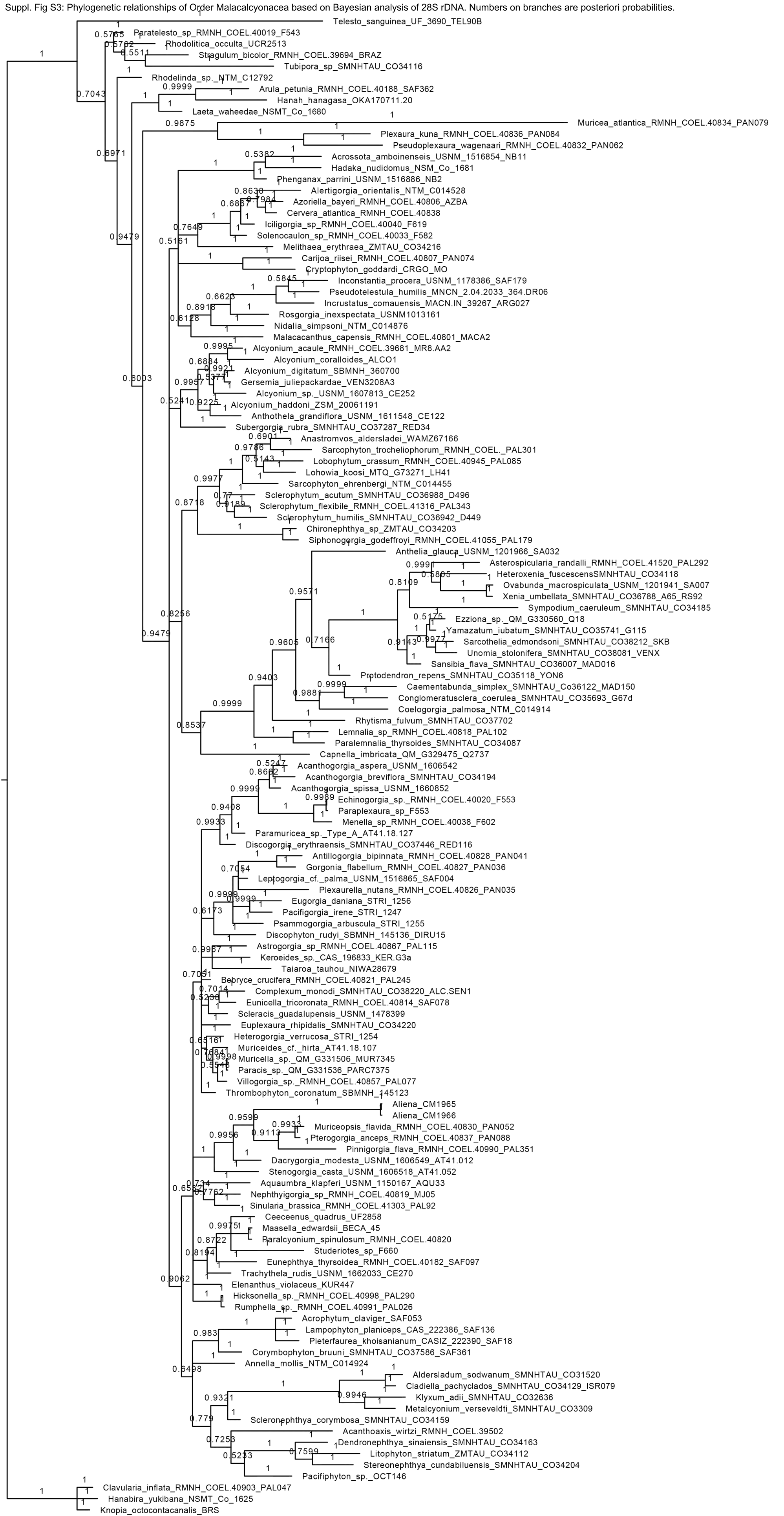

0.08

Supplement: Supplementary material 4 — Phylogenetic relationships of Order Malacalcyonacea based on Bayesian analysis of 28S rDNA [file zookeys-1169-317_article-100576__-s004.pdf]
